# Supplementary material for: The impact of soil transmitted helminth on malaria clinical presentation and treatment outcome: A case control study among children in Bagamoyo district, coastal region of Tanzania
Source: PLoS Negl Trop Dis. 2024 Aug 12;18(8):e0012412. doi: 10.1371/journal.pntd.0012412 (PMC11341094; doi:10.1371/journal.pntd.0012412)
Supplement: Attachment — Submitted filename: review comments.docx [file pntd.0012412.s003.docx]

**Title: The impact of soil transmitted helminth on malaria clinical presentation and treatment outcome: A case control study among children in Bagamoyo district, coastal region of Tanzania**

General comments

- The article is well designed scientifically and methodologically

Specific comments

On the title:

- There are several studies conducted before, what is novelty and the values that add the previous study?
- In the case of co-infection with malaria and STH infection
  - How do you differentiate whether malaria or STH cause the occurrence of other diseases
- Did you measure the severity of the other diseases?

**Method and materials**

- Please describe the current situation of malaria and STHs in the study area.
- How do you allocate your sample size to the health facilites? You have to mention the names of the hospitals too.

**Sample size determination**

- How did you determine your sample size?

**Sample collection**

- Which sample you did collect for malaria? Is it capillary or finger prick
- Why you tried to assess schistosoma specie and *Wuchereria bancrofti* since these parasites are not STH(line 208&209)
- What is the purpose of doing mRDT and blood film? You can just do only blood film since it is the gold standard

**Variables**

- How do you do control cofounding variables?
- Finally why you waited until this year to publish the article since the study was done in 2011/2012?
