# Supplementary material for: The impact of soil transmitted helminth on malaria clinical presentation and treatment outcome: A case control study among children in Bagamoyo district, coastal region of Tanzania
Source: PLoS Negl Trop Dis. 2024 Aug 12;18(8):e0012412. doi: 10.1371/journal.pntd.0012412 (PMC11341094; doi:10.1371/journal.pntd.0012412)
Supplement: Attachment — Submitted filename: Reviewers responses_02072024.docx [file pntd.0012412.s004.docx]

| **Title: The impact of soil transmitted helminth on malaria clinical presentation and treatment outcome: A case control study among children in Bagamoyo district, coastal region of Tanzania** | | | |
| --- | --- | --- | --- |
| SN | Section | Comment | Responses |
| 1 | **General** | The article is well designed scientifically and methodologically | Thank you, appreciated |
| 2 | **Title** | There are several studies conducted before, what is novelty and the values that add to the previous study? | To our knowledge, this is the first case-control study designed to investigate the impact of STH on malaria clinical presentation, response to treatment and outcome and first report on the effect of *E. vermicularis* and *S. stercoralis* on malaria burden.  It is also the first time that the whole childhood range (2months to 9 years) is covered and the whole spectrum from asymptomatic *Plasmodium* infection to uncomplicated and severe malaria studied.  The utilization of a range of diagnostic tests enabled us to explore a wide range of effect of various species of STH and malaria.  For the first time, we provide highlights for the case of *Plasmodium* and *Enterobius vermicularis* co-infection in Tanzanian children. |
| 3 |  | In the case of co-infection with malaria and STH infection   - How do you differentiate whether malaria or STH cause the occurrence of other diseases | Based on the design of our study, we can’t establish a causal relationship between STH and Malaria. To study causality of malaria or STH Vs other co morbidity requires a larger randomized control study purposefully sized to have enough power.  The results of our study provide the trends and association of effects as explored by various models and statistical tests. We highlight the trend and influence of helminths on parasite clearance and occurrence pattern with other co morbidity. |
| 4 |  | Did you measure the severity of the other diseases? | No, the sample size didn’t allow further exploration in terms of severity of other diseases. This is among our limitations as mentioned in the narration. |
| 5 | **Method and materials** | Please describe the current situation of malaria and STHs in the study area | Thank you for the input. The information has been added under the background section and reference list updated |
| 6 |  | How do you allocate your sample size to the health facilities? You have to mention the names of the hospitals too. | The information on the villages included has been updated under the study area. Number of facilities and more details narrated under the study area.  The sample size was powered based on 10% prevalence of *Plasmodium* parasitemia in the study area aiming to recruit 100 asymptomatic children as controls. One thousand (1,000) children from the community were screened to attain the controls. Four main villages were purposely selected considering the transmission of malaria and helminth and possible children identified by the village health care workers. We didn’t utilize proportionate sampling assuming all the selected villages had the same burden.  The sampling procedures were also explained in previous two published studies referenced in the manuscript.  Salim N, Knopp S, Lweno O, Abdul U, Mohamed A, Schindler T, Rothen J, Masimba J, Kwaba D, Mohammed AS, Althaus F. Distribution and risk factors for Plasmodium and helminth co-infections: a cross-sectional survey among children in Bagamoyo district, coastal region of Tanzania. PLoS neglected tropical diseases. 2015 Apr 2;9(4):e0003660.  Salim N, Schindler T, Abdul U, Rothen J, Genton B, Lweno O, Mohammed AS, Masimba J, Kwaba D, Abdulla S, Tanner M. Enterobiasis and strongyloidiasis and associated co-infections and morbidity markers in infants, preschool-and school-aged children from rural coastal Tanzania: a cross-sectional study. BMC infectious diseases. 2014 Dec;14:1-7. |
| 7 | **Sample size determination** | How did you determine your sample size? | Please refer to above narration. Clarification has been added under participant recruitment. |
| 8 | **Sample collection** | Which sample you did collect for malaria? Is it capillary or finger prick | Finger prick was done and estimated 1.0ml was collected. mRDTs were done at the field to support screening and management of children. Details added under sample collection section. |
| 9 |  | Why you tried to assess schistosoma species and *Wuchereria bancrofti* since these parasites are not STH (line 208&209) | The decision was triggered by the integrated control program, a neglected tropical disease control program (NTDCP) in the country including the National lymphatic filariasis elimination program (NLFEP). To assess the whole range of parasitic burden, other species were included as per District decision based on the burden.  Blood samples from children who entered the study from July 2012 onwards were additionally examined for *Wuchereria bancrofti* antigen using the Binax NOW Filariasis rapid immunochromatic test (ICT)-card (Inverness Medical Professional Diagnostics; Scarborough, Maine, United States of America). |
| 10 |  | What is the purpose of doing mRDT and blood film? You can just do only blood film since it is the gold standard | Thin and thick blood films were performed, air dried and Giemsa stained for detection and quantification of malaria parasites.  mRDTs were conducted for the purpose of screening and decision to manage the children in the field. |
| 11 | **Variables** | How do you do control cofounding variables? | Confounding was controlled in several manners in this study.  i. The design of the study (Case control), ii. Matching iii. Multivariant regression analysis iv. Exploration using various models |
| 12 |  | Finally, why you waited until this year to publish the article since the study was done in 2011/2012? | Our apologies to the scientific contribution, the reasons were beyond our control, we aimed to publish the work through other forum and later our colleague moved to another institution. This contributed to the delay in compilation of the work.  We will appreciate swift approval and sharing results to the scientific world. |
